# A novel *ELP1* mutation impairs the function of the Elongator complex and causes a severe neurodevelopmental phenotype

**DOI:** 10.1038/s10038-023-01135-3

**Published:** 2023-03-02

**Authors:** Marija Kojic, Nour E. H. Abbassi, Ting-Yu Lin, Alun Jones, Emma L. Wakeling, Emma Clement, Vasiliki Nakou, Matthew Singleton, Dominika Dobosz, Marios Kaliakatsos, Sebastian Glatt, Brandon J. Wainwright

**Affiliations:** 1 Frazer Institute, The University of Queensland, Woolloongabba, QLD 4102 Australia; 2 Malopolska Centre of Biotechnology, Jagiellonian University, Krakow, Poland; 3 Postgraduate School of Molecular Medicine, Medical University of Warsaw, 02-091 Warsaw, Poland; 4 Institute for Molecular Bioscience, The University of Queensland, Brisbane, QLD Australia; 5 North East Thames Regional Genetic Service, Great Ormond Street Hospital for Children NHS Foundation Trust, London, UK; 6 Paediatric Neurology, Great Ormond Street Hospital for Children NHS Foundation Trust/ University College London, London, UK

**Keywords:** Neurodegeneration, Disease genetics

## Abstract

**Background:**

Neurodevelopmental disorders (NDDs) are heterogeneous, debilitating conditions that include motor and cognitive disability and social deficits. The genetic factors underlying the complex phenotype of NDDs remain to be elucidated. Accumulating evidence suggest that the Elongator complex plays a role in NDDs, given that patient-derived mutations in its ELP2, ELP3, ELP4 and ELP6 subunits have been associated with these disorders. Pathogenic variants in its largest subunit ELP1 have been previously found in familial dysautonomia and medulloblastoma, with no link to NDDs affecting primarily the central nervous system.

**Methods:**

Clinical investigation included patient history and physical, neurological and magnetic resonance imaging (MRI) examination. A novel homozygous likely pathogenic *ELP1* variant was identified by whole-genome sequencing. Functional studies included in silico analysis of the mutated ELP1 in the context of the holo-complex, production and purification of the ELP1 harbouring the identified mutation and in vitro analyses using microscale thermophoresis for tRNA binding assay and acetyl-CoA hydrolysis assay. Patient fibroblasts were harvested for tRNA modification analysis using HPLC coupled to mass spectrometry.

**Results:**

We report a novel missense mutation in the *ELP1* identified in two siblings with intellectual disability and global developmental delay. We show that the mutation perturbs the ability of ELP123 to bind tRNAs and compromises the function of the Elongator in vitro and in human cells.

**Conclusion:**

Our study expands the mutational spectrum of *ELP1* and its association with different neurodevelopmental conditions and provides a specific target for genetic counselling.

## Introduction

With the advent of rapid and affordable whole genome/exome analysis, the traditional human genetic paradigm has been somewhat inverted. Whereas previously a genetic syndrome was initially defined clinically, and then causative genes were discovered, nowadays gene mutations are identified first which then allows the definition of a broad clinical spectrum beyond the index patient. This is particularly true in neurodevelopmental disorders (NDDs) where a great deal of phenotypic variation yet may show evidence of single gene inheritance. Neurodevelopmental disorders are a group of disorders that impede brain development and are characterized by impaired learning, motor, social and occupational functioning. They affect 2–5% of children [1] and are clinically and genetically variable. Despite a number of NDD-causative genes being identified, most patients do not currently receive a molecular diagnosis due to the heterogeneity and/or a polygenic nature of the disorders [2]. Nonetheless, there is a growing body of evidence showing that mutations in subunits of the Elongator complex lead to severe neurodevelopmental phenotypes [3–14].

The dodecameric Elongator complex consists of two subcomplexes, the catalytic ELP123 and accessory ELP456 with two copies of each of the subunits (ELP1-6) [15]. The complex plays a critical role in translation by modifying uridines in the wobble position (U_34_) to 5-carboxymethyl-uridine (cm^5^U_34_) in the anticodon of 12 tRNA species in mammals [16, 17]. This modification is further converted to 5-carbamoylmethyl-uridine (ncm^5^U_34_), 5-methoxycarbonylmethyl-uridine (mcm^5^U_34_) and 5-methoxycarbonymethyl-2-thiouridine (mcm^5^s^2^U_34_) [18]. ELP1 is the largest subunit of the complex and acts as the major scaffolding platform for the other subunits. Therefore, ELP1 is essential for the assembly and the stability of the holo-complex [19]. Variants in the catalytic subunit of the complex, *ELP3*, have been implicated by GWAS studies in amyotrophic lateral sclerosis [6, 7], whilst causative mutations in the *ELP2* gene are found in the patients with NDDs, including intellectual disability (ID), autism and epilepsy [4, 8–10]. In addition, NDD-causing variants have also been reported in the Elongator accessory sub-complex, with ELP4 [11–14] and ELP6 [5] affected. All reported disease-causing Elongator variants have been found to be germline either homozygous hypomorphic missense mutations or compound heterozygotes consisting of one loss-of-function (LoF) allele and another allele harbouring a pathogenic missense mutation. However, the mutations still permit the expression of the respective proteins at certain levels, as a complete loss of any of the Elongator subunits is embryonically lethal in mammals [4, 20–22].

Studies of a predominantly autonomic nervous system (ANS) disorder, familial dysautonomia (FD), have identified a missense mutation in the *ELP1* gene resulting in an exome skipping event leading to the tissue-specific manner depletion of the gene product [23–25]. In addition to the ANS, FD has also detrimental effects on the central nervous system (CNS) development, which is similar to other Elongator mutations. The CNS-related clinical features include visual [26, 27] and learning impairment [28–30], seizures, reduced motor nerve conduction, brain stem reflexes deficits [31] and white and grey matter microstructural lesions evident on magnetic resonance imaging (MRI) [32]. *ELP1* was also found to be the most common medulloblastoma predisposition gene as a number of germline LoF mutations were identified in the patients with this type of pediatric brain cancer [33]. The tumours are characterized by biallelic inactivation of ELP1 due to somatic loss of chromosome arm 9q. In clear distinction from previous work, where reduced ELP1 protein levels result in FD and a complete loss of ELP1 in granule neurons actually promotes tumorigenesis, we report a single amino acid substitution in *ELP1*, namely *ELP1K815T*, that causes a severe neurodevelopmental phenotype in two siblings. This is the first association of ELP1 with NDDs primarily affecting the CNS. Here, we performed functional in vitro and in vivo studies to define the consequences of the mutation on the function of Elongator and determined that the *ELP1K815T* mutation reduced tRNA binding affinity and catalytic activity of the complex leading to aberrant tRNA modification profiles. We also demonstrate that patient-derived *ELP1K815T* fibroblasts could be readily established to study Elongator-specific tRNA modification defects, showing that for future patients and their families, Elongator function can be measured robustly, facilitating a genotype/phenotype relationship to be established for the purpose of clinical counselling.

## Materials and methods

### Clinical data collection

Patients with the homozygous *ELP1* variant were recruited from Great Ormond Street Hospital in the United Kingdom. The patients were clinically assessed by pediatric neurologists and medical geneticists. Patient 2 underwent a skin biopsy as a part of the clinical diagnostic investigation and skin fibroblasts were obtained and cultured for functional analyses.

### Whole-genome sequencing

Whole-genome sequencing was carried out via the 100,000 Genomes Project [34]. A number of standardized panels from the Genomics England PanelApp were subsequently applied to the data (https://panelapp.genomicsengland.co.uk/) and a review of prioritized variants outside the panels was also undertaken. Targeted sequence analysis by bidirectional Sanger sequencing was used to confirm the presence of the variant in both siblings. Full informed consent was obtained to participate in the 100,000 Genomes Project.

### DNA constructs

Codon-optimized open reading frames of ELP1 (O95163), ELP2 (Q61A86) and ELP3 (Q9H9T3) from *Homo sapiens* were cloned into pFastBac1 HTa. ELP3 was cloned with an additional in-frame Twin-strep-Tag at its 3ʹ end. The ELP123 construct was generated using Gibson assembly [35] by amplifying all three genes in PCR with primers adding specific overhangs on 5ʹ and 3ʹ sides of each expression cassette, allowing to determine the specific order of the ORFs. Subsequently, the amplified modules were assembled within the pBiG1a plasmid using established protocols and primers. Mutations in ELP123 were introduced by QuikChange mutagenesis. The construct for production of the ELP456 complex from *Homo sapiens* in insect cells was previously described [5]. As for the human ELP1 plasmid construction, a pair of primers were designed to amplify the region between amino acid 715 to 1332 with NcoI and XhoI sites. The PCR products were treated with NcoI and XhoI and cleaned using a DNA extraction kit. The double digested DNA fragments were then cloned into pETM11 vector.

### Recombinant protein production and purification

For ELP123 protein expression, SuperSf9-3 cells were infected with multiplicity of infection (MOI) = 1 and grown for 3 days at 27 °C on a shaking platform. Subsequently, insect cells were lysed in Lysis Buffer (for ELP123: 50 mM HEPES pH 7.5, 100 mM NaCl, 2 mM DTT, 5% glycerol, DNase I, protease inhibitors; for ELP456: 50 mM HEPES pH 7.5, 150 mM NaCl, 2 mM MgCl_2_, 2 mM DTT, 5% glycerol, 10 mM imidazole, DNase I, protease inhibitors) by 3 cycles of freezing and thawing in liquid nitrogen and sonication, followed by two-step centrifugation (4 °C; 1 h; 80,000 × *g*). ELP123 variants were purified using StrepTrap HP 5 ml column (GE Healthcare) eluted in Strep Elution Buffer (50 mM HEPES, 100 mM NaCl, 1 mM DTT, 5% glycerol, 10 mM d-desthiobiotin, pH 7.5), followed by affinity chromatography on HiTrap Heparin HP 5 ml column (GE Healthcare) eluted in a gradient of Heparin Elution Buffer (50 mM HEPES, 1 M KCl, 1 mM DTT, pH 7.5). Finally, eluates were run on Superose 6 Increase 10/300 GL column (GE Healthcare) in 20 mM HEPES pH 7.5, 100 mM NaCl, 5 mM DTT. ELP456 supernatants were purified on IgG agarose beads (Merck) followed by overnight Tobacco Etch Virus (TEV) protease cleavage in Cleavage Buffer (50 mM HEPES pH 7.5, 150 mM NaCl, 2 mM MgCl_2_, 2 mM DTT). On the next day, the protein sample was applied to a S200 Increase 10/300 GL column (GE Healthcare) equilibrated in 20 mM HEPES pH 7.5, 100 mM NaCl, 2 mM MgCl_2_, 5 mM DTT. Selected fractions were pooled and concentrated with on an Amicon Ultra-0.5 (100 kDa cut-off) concentrator. Aliquots were frozen in liquid nitrogen and stored at −80 °C for further use.

For human ELP1_715-1332_ production, the plasmids were transformed into BL21 pRARE cells. The purification of ELP1CT has been described previously [19]. In details, the cells were inoculated and cultured in LB at 37 °C. IPTG (0.3 M) was added until the OD_600_ reached 0.6 and the cell culture was then incubated at 16 °C for 18 h with shaking. The cells were then collected and lysed in lysis buffer (50 mM Tris pH 7.5, 100 mM NaCl, 20 mM imidazole, 1 mM MgCl_2_, 2 mM B-ME 10% glycerol). The debris was removed by centrifugation and the protein containing supernatant was mixed with NiNTA resin for target protein purification. The binding was performed at 4 °C for 1 h and the beads were washed by washing buffer (50 mM Tris pH 7.5, 100 mM NaCl, 200 mM KCl, 20 mM imidazole, 1 mM MgCl_2_, 2 mM B-ME 10% glycerol). The bound protein fraction was eluted with elution buffer (50 mM Tris pH 7.5, 100 mM NaCl, 8 mM imidazole, 1 mM MgCl_2_, 2 mM B-ME 10% glycerol). The elute was further loaded onto a S200 10/300 increase gel filtration column for obtaining a homogenous sample. Selected fractions were pooled and concentrated with on an Amicon Ultra-0.5 (30 kDa cut-off) concentrator. Aliquots were frozen in liquid nitrogen and stored at −80 °C for further use.

### nanoDSF

ELP1 proteins were prepared at 1 g/l concentration in 50 mM Tris pH 7.5, 100 mM NaCl, 2 mM DTT and loaded into capillaries. The changes of intrinsic fluorescence of the target protein upon heating were monitored using the PROMETHEUS PANTA (Nanotemper Technologies) and the unfolding profiles were determined by MO-analysis from at least three independent experiments (Nanotemper Technologies). The statistical analysis (*p* value) of results from triplicates was performed using *t* test (two-tails).

### ELP123456 in vitro pull-down

For pull-down experiments, the ELP123_WT_ subcomplexes were immobilized on the Dynabeads MyOne Streptavidin C1 resin (ThermoFischer) via the Twin-Strep-tagged ELP3 protein and incubated with the ELP456 variants for 30 min at 4 °C, washed 3 times in Wash Buffer (20 mM HEPES pH 7.5, 100 mM NaCl, 1 mM DTT, 0.05% Tween 20).

The ELP456_WT_ subcomplexes were immobilized on Anti-DYKDDDDK (PierceTM) resin via FLAG-tagged ELP6, incubated for 30 min at 4 °C and washed three times in Wash Buffer (same as for Streptactin pull-down). For both pull downs, the proteins were liberated from the beads by heating the sample in SB for 5 min at 95 °C before loading the inputs and pull-downs on the SDS-PAGE gels and visualizing by Coomassie staining. Band intensities were quantified using ImageJ and the statistical analysis (*p* value) from triplicates was performed using *t* test (two-tails).

### Structure modelling

The predicted human ELP1, ELP2 and ELP3 protein atomic models were obtained from the online prediction software Alphafold2 (AF-O95163-F1, AF-Q61A86-F1, AF-Q9H9T3-F1). The individual model was fitted into the yeast Elongator 123 density (PDB: 6qk7) using Chimera (version 1.2). The tRNA (PDB: 1ehz) was taken from the Protein Data Bank and fit into the human Elongator based on the yeast tRNA fit. The analysis of ELP1 Lys815 was performed using the density and model of the ELP1 C-terminus (PDB: 5CQR) solved by crystallography. The analysis and figures were performed using Pymol, the Molecular graphics system (version 2.0 Schrödinger, LLC).

### In vitro-transcribed tRNAs

The tRNA was produced using the T7 RNA polymerase-mediated run off method [36]. The DNA template contained a T7-promoter sequence and followed by the tRNA^Gln^_UUG_ sequence. The in vitro transcription reaction was performed in a 500 µL volume containing DNA template, T7 RNA polymerase and reaction buffer (20 mM Tris, pH 8.0, 5 mM DTT, 150 mM NaCl, 8 mM MgCl_2_, 2 mM spermidine, 20 mM NTPs, RNasin, and pyrophosphatase). The reaction was performed at 37 °C for overnight and followed by DNaseI treatment to remove DNA templates. The product was then purified using a DEAE column and heat treatment at 80 °C for 2 min and followed by slow cooling process to room temperature as the re-annealing process. To obtain a homogenous tRNA population, the samples was subjected to a S75 Increase gel filtration column and the tRNA containing fractions were pooled and stored at -80 °C. For MST assays, the internally Cy5-labelled in vitro-transcribed human tRNA^Gln^_UUG_ was produced as mentioned above where the additional 5% of Cy5-CTP was introduced in the reaction.

### Acetyl-CoA hydrolysis assay

Purified ELP123 (0.475 μM) was mixed with 10 μM in vitro-transcribed tRNA^Gln^_UUG_ in presence of 500 μM acetyl-CoA in 1x acetyl-CoA Assay Buffer (MAK039, Sigma) and incubated in a thermocycler for 30 min at 37 °C. To remove proteins and tRNAs, the samples were passed through a 3 kDa cut-off concentrator (EMD Millipore). The flow-through was collected and subjected to an acetyl-CoA assay kit (MAK039, Merck) for quantitation determinations. The reactions were performed according to the manufacturer’s instructions. Fluorescence intensity was measured using a plate reader (TECAN) at the probe-specific excitation (535 nm) and emission (587 nm) wavelengths. The measurements for individual conditions were calculated from at least three independent experiments. The graphs were prepared using Prism v8.0.2 (GraphPad) software.

### Microscale thermophoresis (MST) for tRNA binding assay

The Cy5-labelled tRNA^Gln^_UUG_ (14 nM) was incubated with serial dilutions of purified ELP123 variants (starting from 1.5 μM) in MST Buffer (20 mM HEPES, 100 mM NaCl, 5 mM DTT, pH 7.5, 0.05% Tween 20) at 4 °C for 30 min. The samples were applied to capillaries (MO-K025, Nanotemper Technologies) and the measurements were performed using Monolith Pico (Nanotemper Technologies) with 60% excitation power at 25 °C. Obtained data were analyzed and dissociation constant values were calculated using MO.2 Affinity software (Nanotemper Technologies) from at least three independent repeats. The graphs were prepared using Prism v8.0.2 (GraphPad) software.

### Fibroblast culture

Patient fibroblasts were obtained via punch biopsy and control fibroblasts (TIG-102) were purchased from JCRB Cell Bank. The cells were cultured at 37 °C with 5% CO_2_ in Dulbecco’s Modified Eagle’s Medium (11995065, ThermoFisher) with 10% FBS (10099141, ThermoFisher) and added penicillin/streptomycin (15070063, ThermoFisher). The cells were harvested, diluted, and plated to obtain single cell-derived clones. The clones were expanded in culture for two additional passages and dissociated by trypsinization (25200072, ThermoFisher). All centrifugation steps were performed at 1000 × *g* for 5 min. Approximately 2 million cells were harvested for tRNA modification analyses.

### tRNA modification analyses

Fibroblasts were homogenized in a TRIzol reagent (Life Technologies) using tissue homogenizer (Bertin Technologies). Total RNA and tRNA extraction, tRNA hydrolysis to ribonucleosides and tRNA modification analysis were performed as previously described [4].

High-performance liquid chromatography coupled to mass spectrometry was performed using a Luna Omega 1.6 μm, Polar-C18 100 Å column (150 mm × 2.1 mm, Phenomenex, Australia). Mass spectrometry parameters were determined for the ribonucleosides using multiple injections of 0.1–1 ng of purified ncm^5^U, mcm^5^U and mcm^5^s^2^U nucleosides (a generous gift from Sebastian Leidel, University of Bern, Switzerland) and commercially obtained m^7^G (Santa Cruz Biotechnology). Retention times were determined based on the available compounds (ncm^5^U, mcm^5^U, mcm^5^s^2^U and m^7^G) and for m^1^A based on the previously published results [37]. Peak assignment and quantification were performed using MultiQuant-v2.1.1 (ABSciex) software. Pseudouridine (Ψ) was used to normalize the data. Statistical analysis was performed using Prism v9.4.0 (GraphPad) software. Number of replicates, the statistical test and statistically significant differences are indicated in the figure legend. Differences between groups were considered statistically significant for *p*  ≤  0.05.

### RNA extraction, reverse transcription cDNA synthesis and qPCR

Total RNA was extracted from the patient’s and control fibroblasts (2.5 × 10^6^ cells per genotype) using RNaesy Mini Kit (Qiagen, 74904) according to the manufacturer’s protocol. On-column DNA digestion was performed using RNase-Free DNase Set (Qiagen, 79254). First-strand cDNA was synthesized from 1 μg of total RNA with oligo(dT) hexamers using SuperScript™ III First-Strand Synthesis System (ThermoFisher, 18080051). cDNA was stored at −20 °C for future use. For qPCR analysis, each cDNA sample was diluted 10 times with nuclease-free water. Quantitative PCR (qPCR) analysis was performed using Quantstudio^TM^ 7 Flex system (Applied Biosystems). Each 10 μl reaction contained 40 ng of cDNA, qPCR Master Mix (ThermoFisher, 4444556) and ELP1 (FAM, HS00175353_m1) and GAPDH (VIC, HS02786624_g1) TaqMan probes. The amplification program was as follows: 95 °C for 10 min, 40 cycles at 95 °C for 15 s and 60 °C for 1 min. The expression of *ELP1* mRNA was normalized to *GAPDH* expression. Number of replicates, the statistical test and statistically significant differences are indicated in the figure legend.

## Results

### *ELP1K815T* variant causes developmental delay and intellectual disability

**Fig. 1:**
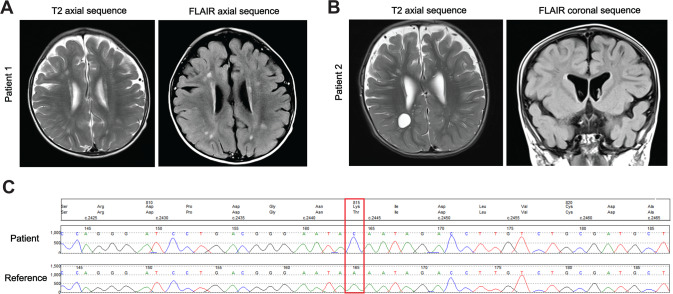
MRI findings and whole-genome sequencing of the patients carrying disease-causing *ELP1* variant. **A** MRI brain scans of Patient 1 showing non-specific white matter lesions scattered throughout the cerebral hemisphere and (**B**) patient 2 showing prominent perivascular spaces. **C** Whole-genome sequencing chromatogram with the identified variant *ELP1K815T* indicated by the red rectangle

We identified two siblings with a complex neurodevelopmental phenotype in our routine clinical examination. Phenotypic characteristics and clinical descriptions of the patients are summarized in Table 1. Both siblings were non-verbal and had a severe global developmental delay and intellectual disability. Magnetic resonance imaging revealed white matter lesions with enlarged perivascular spaces (Fig. 1A, B). This was suggestive of an inflammatory reaction associated with demyelination. Whole-genome sequencing identified a homozygous variant in the *ELP1* gene, c.2444 A > C; p.Lys815Thr (*ELP1K815T*) in both siblings (Fig. 1C). The variant was absent from gnomAD database of genetic variation. The parents were first cousins, showed no clinical symptoms and were found to be heterozygous carriers of the *ELP1*. We set out to perform in vitro and in vivo analyses to characterize the mutation and explore its consequences at the molecular level.

**Table 1:** Clinical data of the patients carrying disease-causing *ELP1* variant

	Patient 1	Patient 2
Elongator variants	ELP1 c.2444A>C; p.Lys815Thr	ELP1 c.2444A>C; p.Lys815Thr
Sex/age	Female/9 y 9 m	Male/7 y 10 m
Prenatal/neonatal course	Foetal distress/Uncomplicated	Uncomplicated
Developmental delay	Severe	Severe
Intellectual disability	Severe	Severe
Age of sitting/walking	13 m/3 y	18 m/7 y with support
Language abilities	Non-verbal, babbles and points	Non-verbal, babbles
Fine motor skills	Delayed, at 3.5 y developed pincer grip, assistance required to hold a pencil, she can finger feed herself and use a spoon	Delayed, at 2 y not able to reach out for toys or transfer from hand to hand, he can finger feed himself and use a spoon
Vision and hearing	Normal	Normal
Brain MRI	Non-specific white matter lesions scattered throughout the cerebral hemisphere white matter, reduced volume of white matter with prominent perivascular spaces	Underdevelopment of cerebral white matter with prominent perivascular spaces
Clinical features	Hypermobile, head circumference on the 4th centile	Hypermobile, head circumference on the 14th centile

*m* months, *MRI* magnetic resonance imaging, *y* years

### *ELP1K815T* does not affect the integrity of ELP123 or Elongator assembly

**Fig. 2:**
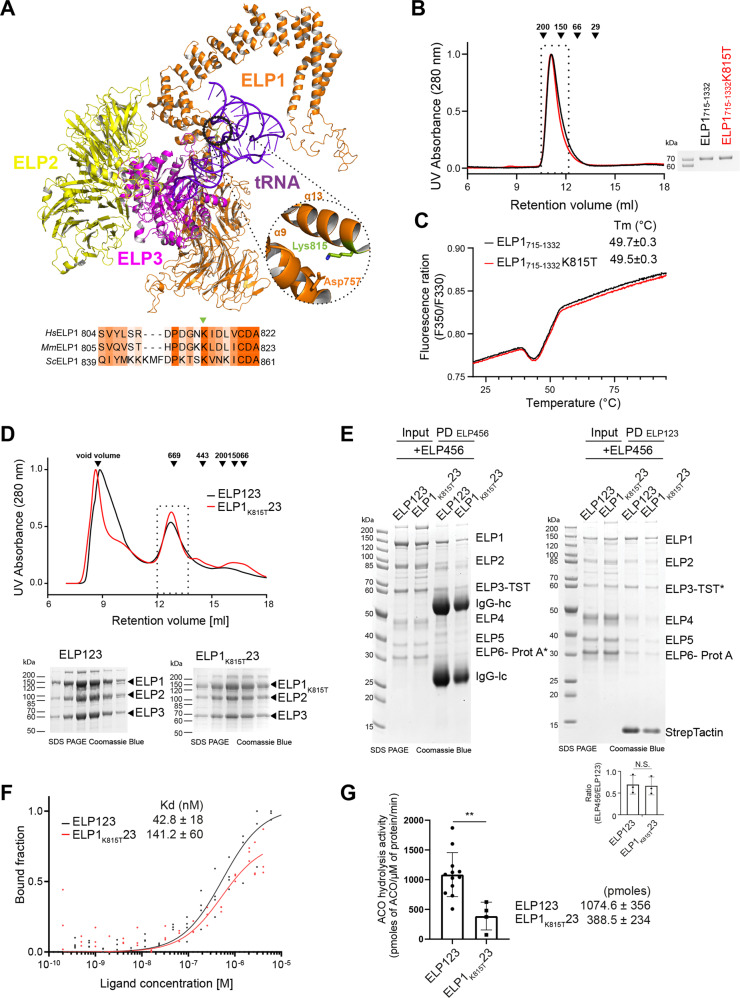
Biochemical characterization of recombinant ELP1K815T23 in vitro. **A** Localization of patient-derived ELP1K815T residue in the ELP123 model (ELP123; AF-O95163_F1, AF_Q61A86, AF_Q9H9T3_F1) which is based on the cryo-EM structure of yeast ELP123 (yELP123; PDB 6qk7). The subunits are colour-coded: ELP1-orange, ELP2-yellow, ELP3-magenta and tRNA is in purple (PDB 1EHZ). The K815T (green) is located in the tetratricopeptide (TPR) region of the ELP1. A close-up view at the K815 residue in the ELP1 crystal structure (PDB 5cqr) and the interacting residue (D757) is shown. A schematic presentation of the partial sequence alignment of several eukaryotic Elp1 proteins. The residues in the proximity of the K815 (indicated by a triangle) are shown. **B** Size-exclusion chromatography profiles of human ELP1_715-1332_ and ELP1_715-1332_K815T. Calibration standards are shown as black arrows indicating the respective molecular mass in kDa. The eluted fractions were resolved in a 12% SDS-PAGE and visualized by Coomassie staining. **C** Thermal shift measurements of ELP1_715-1332_ and ELP1_715-1332_K815T are shown and the calculated Tm (°C) are shown (*n* ≥ 3). **D** Size-exclusion chromatography profiles of ELP123 and ELP1_K815T_23. The standards to calibrate the column appear in kDa above the black arrows. The eluted fractions were resolved in a 12% SDS-PAGE and visualized by Coomassie staining. **E** Pull-down analyses of the assembled Elongator holo-complexes immobilized on IgG beads via ELP6 protein (ELP6-Prot A; left) and on StrepTactin beads via ELP3 protein (ELP3-TST; right) and after incubation with the respective complementing subcomplexes, ELP456 (left) and ELP123 (right). The quantified intensities of ELP123 and ELP456 complexes from three independent StrepTactin-mediated pull down are shown at the bottom of the gel. Intensities were normalized to StrepTactin bands (*n* ≥ 3). **F** tRNA binding analysis of ELP123 proteins for human tRNA_Gln_^UCG^ by microscale thermophoresis. *n* = 3 technical repeats. Average Kd (nM) values are included in the plot. **G** Acetyl-CoA hydrolysis assay of purified ELP123 in the presence of tRNA_Gln_^UCG^. *n* > 3 technical repeats. Statistical analysis: *t* test. Statistically significant differences are indicated (**p  ≤  0.05). Data represent mean ± SEM

To study the impact of the mutation on the function of the complex, we created in silico models of ELP1, ELP2 and ELP3 using Alphafold 2 (AF2; https://alphafold.ebi.ac.uk/) and swissmodel (https://swissmodel.expasy.org/), respectively. By superimposing the models of the individual subunits and the previously determined crystal structure of the ELP1 C-terminal domain (CTD, 5cqr) on our previous yeast Elp123 cryo-EM model (6qk7), we created a model of the ELP123 sub-complex (Fig. 2A). Sequence alignment analysis of ELP1 proteins showed that Lys815 is highly conserved across eukaryotes, which further highlights the importance of the residue (Fig. 2A). We used the high-confidence model to locate the mutated residue, which is situated in the base of the tetratricopeptide repeat (TPR) domain. The position of Lys815 is not in the proximity of any structural motif involved in the catalytic activity of the complex (e.g. acetyl-CoA binding site of ELP3, the dimer interface of the ELP1 or ATPase sites in ELP5 and ELP6). Based on the previously resolved ELP1 crystal structure [19], we further analyzed the available density of the structure and observed that the side chain of Lys815 forms a direct contact with Asp757, spanning from helix 9 to helix 13 in the predicted human model which corresponds to the helices 4 and 7 in the previously obtained structure of yeast Elongator [38]. This indicates that the residue Lys815 is likely involved in maintaining the tertiary structure of ELP1 and any perturbation in this region could affect the local stability and general flexibility of the ELP1 arm.

We also investigated whether the ELP1K815T mutation affects the thermostability of human recombinantly expressed ELP1 in vitro. Due to difficulty in obtaining the full length human ELP1 protein, we produced a N-terminal truncation version of ELP1 (ELP1_715-1332_) that was previously described [19]. Of note, the K815T mutation does not negatively impact the protein production or the known dimerization of the C-terminal construct (Fig. 2B). We then performed the thermal shift assay using differential scanning fluorimetry (DSF) to compare the protein stability of the purified samples (Fig. 2C). Both, ELP1_715-1332_ and ELP1_715-1332_K815T, exhibit similar unfolding profiles and Tm values (49.7 ± 0.3 and 49.5 ± 0.3 °C). Altogether, the amino acid substitution of lysine 815 to threonine does not affect the stability of ELP1 in vitro.

Next, we decided to directly test if the K815T mutation indeed influences the stability and integrity of purified ELP123. Therefore, we employed the BiGBac system [35] to simultaneously produce all three subunits of full-length ELP123 and ELP1_K815T_23 sub-complexes in Super-Sf9-3 insect cells. Consequently, we obtained purified and stoichiometric samples of the wild-type and mutated ELP123 sub-complexes. Both ELP123 and ELP1_K815T_23 eluted at an estimated molecular weight of ~680 kDa, showing no signs of proteolytic degradation and resulted in similar quantities (Fig. 2D). Therefore, the *ELP1* mutation neither affects dimerization of ELP123, nor leads to a dramatic destabilization of the sub-complex during the purification. Furthermore, we used protein-protein interaction assays to test whether the mutation reduced the ability of ELP123 to bind the ELP456 subcomplex. In detail, we performed a series of complementary pull-down experiments using affinity tags in either of the sub-complex. The results clearly show that the K815T mutation did not influence the formation of the fully assembled Elongator complex (Fig. 2E). In summary, our analyses show that the presence of K815T in ELP123 does not negatively influence the formation and integrity of the bi-lobed Elongator complex in vitro.

### *ELP1K815T* causes reduced tRNA binding and catalytic activity of ELP123

On the one hand ELP1 acts as a scaffold for the other Elongator subunits, but on the other hand the ELP1 is also involved in efficient binding of the tRNA substrate, which is clamped between the active site in ELP3 and the CTD of ELP1. To check the effect of the K815T mutation on Elongator’s ability to bind tRNAs and its tRNA modification activity, we used the purified wild-type and mutated subcomplexes in established biochemical assays [4, 36]. First, we used microscale thermophoresis (MST) to test the affinity of ELP123 and ELP1_K815T_23 towards human tRNA^Gln^_UUG_. The ELP123 complex showed a K_D_ of approximately 50 nM, whilst ELP1_K815T_23 reduced the affinity 3-times to an approximate K_D_ of 150 nM (Fig. 2F). Of note, the mutation is located in proximity to the bound tRNA molecule, but we do not believe that the mutation precludes a direct contact between the conserved Lys815 residue and the tRNA. We rather speculate that the mutation weakens the stable conformation of the ELP1 CTD and thereby indirectly reduces the affinity of the tRNA. Our results indicate a restricted access of the tRNA to the binding pocket of the ELP3 caused by a collapse in the ELP1 arm due to the loss of the interaction between Lys815 and Asp757 that influences the flexibility of the ELP1 CTD. It is worth noticing that the binding curve for ELP1_K815T_23 did not reach the plateau, which usually happens if the equilibrium of bound and unbound states has not been accomplished. As we were not able to reach higher concentrations of the mutated sub-complex, it is most likely that we overestimate the K_D_ for the ELP1_K815T_23 mutant and that the actual affinity is even lower than calculated one after fitting.

Furthermore, we set out to explore whether the K815T mutation affected the tRNA-induced acetyl-CoA activity of the catalytic subunit ELP3 [36, 39, 40] in the context of the ELP123 sub-complex. The results of the analyses revealed a significantly lower hydrolysis rate in Elp1_K815T_23 than in the wild-type sub-complex (Fig. 2G). Of note, the assays were performed at rather high concentration of tRNA (2 µM) to compensate for the lower binding constant in the mutant. Therefore, we conclude that the mutation in ELP1 not only reduces substrate binding, but also affects the initial steps of acetyl-CoA hydrolysis. In summary, our results show that the identified single amino acid substitution in ELP1 leaving the complex intact, but specifically affects the tRNA binding and modification activity of the Elongator.

### *ELP1K815T* impairs the function of the complex in vivo

**Fig. 3:**
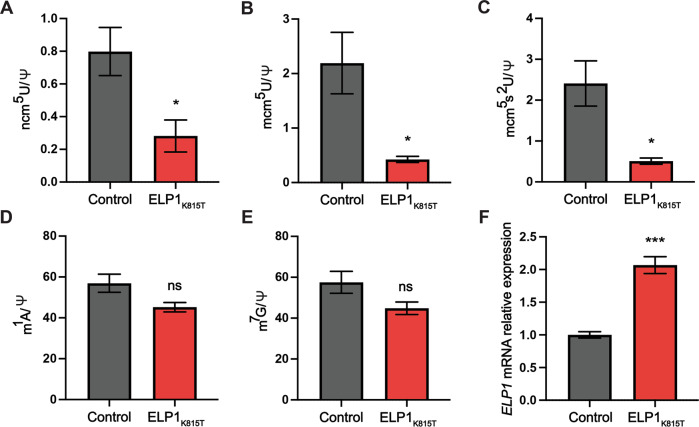
tRNA modification deficiency in fibroblasts derived from the patient with ELP1K815T variant relative to control. High-performance liquid chromatography (HPLC) coupled to mass spectrometry (MS) used to quantify the Elongator-dependent tRNA modifications (**A**) 5-carbamoylmethyluridine (ncm^5^U), (**B**) 5-methoxy-carbonylmethyluridine (mcm5U) and (**C**) 5-methoxycarbonylmethyl-2-thiouridine (mcm5s2U) in human fibroblasts. HPLC-MS was used to assess Elongator-independent modifications (**D**) 1-methyladenosine (m^1^A) and (**E**) 7-methylguanosine (m^7^G) in the cells. Pseudouridine (Ψ) was used as an internal normalization standard. **F** RT-qPCR analysis of *ELP1* relative to *GAPDH* expression in the patient’s and control fibroblasts. *n*  =  5 technical repeats per genotype. Statistical analysis: unpaired two-tailed *t* test (α  =  0.05) with Welch’s correction. Statistically significant differences are indicated (*p  ≤  0.05; ***p  ≤  0.001; ns, not significant). Data represent mean ± SEM

We further investigated the impact of the *ELP1K815T* on the function of the holo-complex in vivo by harvesting fibroblasts of one of the patients and analyzing tRNA modifications affected by the complex. First, we extracted tRNA from the patient’s cells and commercially available fibroblasts from a healthy individual as a control. We then hydrolyzed tRNA to nucleosides and analyzed the modifications using high-performance liquid chromatography coupled to mass spectrometry. The analysis showed that both cm^5^U-dependent modifications, ncm^5^U and mcm^5^U, were markedly reduced in the cells originating from the patient relative to control (Fig. 3A, B). Further modified mcm^5^s^2^U was also significantly affected by the mutation (Fig. 3C).

To determine whether only Elongator-specific modifications were affected, and this was not a tRNA broad effect, we quantified the m^1^A and m^7^G tRNA modifications that do not require Elongator complex for their synthesis. We found that indeed cm^5^U-derived modifications were specifically targeted, whilst Elongator-independent modifications were unaffected (Fig. 3D, E). Hence, the identified *ELP1* variant severely compromises the function of the complex in human cells.

As we showed that the ELP1 stability and expression were not compromised by the K815T substitution in vitro, we further explored whether the mutation affects the expression of this gene in vivo. Patient’s fibroblasts were found to have increased *ELP1* expression relative to control based on reverse transcription-quantitative PCR (RT-qPCR) analysis (Fig. 3F), which would likely be a compensatory response attempting to overcome the reduced function of the complex [41].

## Discussion

Identifying genetic causes of complex NDDs is vital for understanding the molecular mechanisms underlying these incurable conditions and for the delineation of a genotype-phenotype correlation. Early molecular diagnosis is essential for genetic counselling, foreseeing future complications and efficient patient management. Some literature suggests that purely monogenic forms of NDDs are the exception [2]. Nonetheless, an emerging number of studies in the last decade have shown that single mutations in Elongator subunits *ELP2*, *ELP3*, *ELP4* and *ELP6* are associated with a range of neurodevelopmental and neurodegenerative conditions [3–14], which is slowly bringing the complex into the main focus in the field.

Mutations in the Elongator’s largest subunit *ELP1* have been characterized in FD [23–25] and medulloblastoma [33], and the data presented here represent the first report of a mutation in this gene found in patients with NDDs that primarily perturb the development of the CNS. Our data are a clear illustration that the mutational spectrum in a single gene such as *ELP1* can lead to distinct and non-overlapping phenotypes ranging from peripheral neuropathy to complex multidimensional NDDs to predisposition to pediatric brain cancer. All the identified mutations impair the function of the complex in translation given diminished tRNA modification levels found in FD [42], medulloblastoma [33] and both *ELP1K815T* patients. The differences in the clinical phenotypes are likely based not only on different tissues being affected at different stages of the neurodevelopment, but also alternations in protein levels of respective Elongator subunits across different tissues and as we have recently shown, different binding affinity of the complex for specific tRNAs [5]. In FD, there is a tissue-specific reduction in splicing efficiency of *ELP1* affecting development and survival of sensory, sympathetic and parasympathetic neurons [24]. Somatic depletion of ELP1 during granule neuron development in combination with constitutive activation of SHH signaling due to PTCH1 LoF induces tumorigenesis [33]. When it comes to germline pathogenic missense variants in this gene, the mechanism of NDD pathogenesis seems to resemble the one previously described for *ELP2* [4], *ELP4* and *ELP6* [5] mutations.

The clinical features and MRI findings of both siblings correspond to those of patients harbouring pathogenic variants in other Elongator subunits [4, 5, 8–10, 12, 13]. This further confirms that the function of the complex is compromised when any of its subunits is affected, given that we have demonstrated that all described mutations perturb the acetyl-CoA activity of the complex in vitro and tRNA modification in vivo. Aberrant neurogenesis and myelination likely underlie intellectual impairment and developmental delay in *ELP1K815T* patients. Interestingly, the patients do not have microcephaly that has been previously described in the *ELP2* [4], *ELP4* and *ELP6* [5] patients. Demyelination pathogenesis in the siblings may be explained by oligodendrocyte degeneration triggered by unfolded protein response (UPR) as a consequence of translational defects due to tRNA modification loss, which has been demonstrated for the *ELP2* mutations [4]. UPR has been also found to perturb neurogenesis upon Elp3LoF [43] and lead to Purkinje neuron (PN) degeneration in mouse models of patient-derived *ELP2* [4] and *ELP6* [5] mutations. Loss of PNs leads to a motor delay and coordination deficits, both of which were found in the *ELP1K815T* patients, but these are microscopic changes that could not be identified by MRI. The finding of prominent perivascular spaces has been described in other neurometabolic/neurogenetic conditions, e.g. mucopolysaccharidoses, Lowe syndrome, hypomelanosis of Ito. The *ELP1* mutation should now be added to the differential diagnosis of this imaging pattern.

To further understand the underlying neuropathology in the *ELP1* patients, explore whether UPR and translational defects attenuate the neurogenic program and cause degeneration in the brain of the patients, mouse models and iPSC-derived brain organoid studies are required. Although different Elongator pathogenic variants share NDD clinical features, we have recently demonstrated by modelling disease-associated *ELP2* and *ELP6* variants in mice that mutations in the two subcomplexes affect different brain cells during development [5]. Hence, detailed studies of the *ELP1* mutations identified herein will further elucidate the differences between the two sub-complexes and identify the vulnerable CNS structures and molecular consequences of the compromised activity of the complex on the brain cells.

In conclusion, our study demonstrates that screening for mutations in the *ELP1* gene may be beneficial in the clinical genetics practice for NDD patients. We functionally characterize the *ELP1K815T* mutation and show that it destabilizes the complex and impairs its activity resulting in the neuropathology previously defined for Elongator mutants that is yet to be investigated for this mutation. More generally, we present a stark example of the challenges in defining single gene disease, diagnosis and prognosis. Mutational pleiotropy can be such that one gene is causative in a number of distinct and unrelated disease presentations and that ultimately a comprehensive functional characterization using a range of model systems, assays and patient materials is required to make the link between genotype and phenotype.

## Data Availability

The *ELP1* variant identified in the patients has been deposited for public access within DECIPHER with accession number 484902. Data generated in the study are available from corresponding authors on reasonable request.
